# *FABP4* and omentin-1 gene expression in epicardial adipose tissue from coronary artery disease patients

**DOI:** 10.1590/1678-4685-GMB-2020-0441

**Published:** 2021-09-29

**Authors:** Valentina V. Miroshnikova, Ekaterina A. Polyakova, Irina A. Pobozheva, Aleksandra A. Panteleeva, Natalia D. Razgildina, Diana A. Kolodina, Olga D. Belyaeva, Olga A. Berkovich, Sofya N. Pchelina, Elena I. Baranova

**Affiliations:** 1 Pavlov First Saint Petersburg State Medical University, St.-Petersburg, Russian Federation.; 2 National Research Center Kurchatov Institute, Petersburg Nuclear Physics Institute, Gatchina, Russian Federation.

**Keywords:** Coronary artery disease, epicardial adipose tissue, subcutaneous adipose tissue, FABP4, omentin-1

## Abstract

Omentin-1 and fatty acid-binding protein 4 (FABP4) are adipose tissue adipokines linked to obesity-associated cardiovascular complications. The aim of this study was to investigate epicardial adipose tissue (EAT) omentin-1 and *FABP4* gene expression in obese and non-obese patients with coronary artery disease (CAD). Omentin-1 and *FABP4* mRNA levels in EAT and paired subcutaneous adipose tissue (SAT) as well as adipokine serum concentrations were assessed in 77 individuals (61 with CAD; 16 without CAD (NCAD)). EAT *FABP4* mRNA level was decreased in obese CAD patients when compared to obese NCAD individuals (p=0.001). SAT *FABP4* mRNA level was decreased in CAD patients compared to NCAD individuals without respect to their obesity status (p=0.001). Omentin-1 mRNA level in EAT and SAT did not differ between the CAD and NCAD groups. These findings suggest that omentin-1 gene expression in adipose tissue is not changed during CAD; downregulated *FABP4* gene expression in SAT is associated with CAD while EAT *FABP4* gene expression is decreased only in obesity-related CAD.

## Introduction

Adipose tissue produces adipokines, and any abnormality in their expression and secretion can play a role in the development of obesity-associated diseases (Ntaios *et al*., 2013; Lau *et al*., 2017). Previous studies have proposed the imbalance in adipokine secretion in obesity, metabolic syndrome, diabetes, hypertension, atherosclerosis and cardiovascular disease (Lau **et al*.*, 2017). Visceral obesity is generally accompanied by an excess volume of epicardial adipose tissue (EAT), a special visceral fat depot with local and systemic effects. EAT surrounds the heart and is in direct contact with the coronary arteries (Fernández-Trasancos *et al*., 2017). As EAT adipokines can influence the coronary circulation via paracrine and vasocrine mechanisms, EAT may participate in the development of atherosclerosis (Takaoka *et al*., 2009; Iacobellis, 2015). EAT volume is associated with atherosclerotic lesions in the coronary arteries and severity of CAD, as well as fatal and nonfatal coronary events in the general population, regardless of the presence of traditional risk factors of cardiovascular disease, such as smoking, obesity, dyslipidemia and diabetes mellitus (Iacobellis, 2015). 

EAT has a unique transcriptome enriched with genes related to inflammation and endothelial dysfunction and may be closely associated with atherosclerosis initiation and progression (Iacobellis, 2015). Patients with CAD exhibit a pronounced pro-inflammatory gene expression profile of EAT as compared with individuals without CAD (Mazurek *et al*., 2003; Cheng *et al*., 2008; Sacks *et al*., 2011). Considered as pro-inflammatory adipocytokine fatty acid binding protein 4 (FABP4) is locally produced by both EAT and macrophages in vascular plaques and may promote development of coronary atherosclerosis (Lee *et al*., 2013; Furuhashi *et al*., 2016). FABP4 is a member of the lipid chaperone family and regulates the intracellular transport of fatty acids (Rodríguez-Calvo *et al*., 2017). Prospective trials have shown that FABP4 plasma level is associated with the development of metabolic syndrome and type 2 diabetes mellitus and predicts cardiovascular events (Xu *et al*., 2007; Ohyama *et al*., 2013; Rodríguez-Calvo **et al*.*, 2017; Höbaus *et al*., 2018). In addition, plasma level of FABP4 is related to elevated cardiovascular mortality in male patients with type 2 diabetes mellitus (Xu **et al*.*, 2007; Rodríguez-Calvo **et al*.,* 2017).

Among anti-inflammatory and anti-atherogenic adipocytokines, omentin-1, also named intelectin-1, is known to be highly expressed in EAT (Fain *et al*., 2008; Gaborit *et al*., 2015). Serum concentration of omentin-1 is decreased in obese patients, and this phenomenon is considered to be related to insulin resistance (de Souza Batista **et al*.*, 2007). Although studies investigating the link between omentin-1 and cardiovascular outcomes, provided contradictory results, a meta-analysis demonstrated the independent and negative association between omentin-1 serum concentration and CAD (Narumi *et al*., 2014; Agasthi *et al*., 2015; Menzel *et al*., 2016; Saely *et al*., 2016; Zhou *et al*., 2017; Xu *et al*., 2018). 

Omentin-1 supplementation and pharmacological inhibition of FABP4 were shown to reduce atherosclerotic plaque formation in an apolipoprotein E (ApoE)-deficient animal model of atherosclerosis (Fernández-Trasancos *et al*., 2017; Rodríguez-Calvo *et al*., 2017). It is hypothesized that omentin-1 and *FABP4* gene expression in EAT may influence CAD development. In the present study we investigated omentin-1 and *FABP4* gene expression in EAT and paired subcutaneous adipose tissue (SAT) samples of patients with CAD and individuals without CAD (NCAD) with or without abdominal obesity.

## Subjects and Methods

### Subjects

The study enrolled 77 patients who underwent cardiac surgery. Coronary angiography was performed in all participants to detect coronary atherosclerosis and prove CAD. Patients were divided into two groups: CAD patients who underwent coronary artery bypass grafting (n = 61) or NCAD subjects (n = 16; control group). Principal criteria for inclusion to the CAD group: 1-2-3-vessel coronary obstruction demonstrated by coronary angiography. Gensini Score was used as a tool for measuring coronary atherosclerosis severity, every atherosclerotic narrowing from 25% to 100% was included in the calculation multiplied by appropriate Gensini ratio depending on segment location and importance (Gensini, 1983; Neeland *et al*., 2012). The NCAD group included patients undergoing open-heart surgery for valvular replacement without stenosis in coronary artery lumen. Clinical characteristics, including demographic data, body weight, height, waist circumference, medical history and medication use, were obtained from the hospital records. Body mass index (BMI) was calculated as weight (kg) divided by square of height (m^2^). Main exclusion criteria were as follows: cancer, chronic obstructive pulmonary disease, liver or renal failure, connective tissue diseases, acute rheumatic fever, infective endocarditis, hypo/hyperthyroidism, brain diseases, alcohol or drug abuse, acute cerebrovascular accident. EAT thickness was measured by echocardiography using GE VIVID 7 Dimension cardiovascular ultrasound system, in front of the right ventricle wall from the parasternal long axis view.

The study protocol corresponds to the Helsinki Declaration and was approved by the ethics committee of Pavlov First Saint-Petersburg State Medical University, St.-Petersburg, Russian Federation. Written informed consent was obtained from each patient before enrollment.

### Blood sample measurements

Fasting venous blood samples were collected in sodium heparin Vacutainers (Becton-Dickinson), and centrifuged for 15 min at 3000×*g*. The serum samples were then stored −80 °C. Biochemical parameters, including baseline levels of fasting glucose and lipids, were measured, and concentrations of serum omentin-1 and FABP-4 were detected using commercially available enzyme-linked immunosorbent assay kits (RayBiotech, USA, and BioVendor, Czech Republic, respectively) following the manufacturer’s instructions. All samples were measured in triplicates.

### Adipose tissue samples

Paired adipose tissue samples were collected by biopsy from approximately the same location in all patients, including EAT (near right coronary artery ostium) and SAT (from the area of chest incision). Tissue samples (average 0.1 g) were separated from any attached connective tissue and blood vessels and stored at -80 ºC for further RNA extraction.

### RNA isolation and quantitative PCR

Total RNA was extracted from samples using RNeasy kit (Qiagen) and its concentration and purity were assessed by calculating the ratio of optical density at 260 and 280 nm wavelengths (OD 260/280). The integrity of RNA was assessed using non-denaturing 1% agarose gel electrophoresis and reflected by the 18S and 28S ribosomal bands. In brief, 2 µg of RNA from each biopsy was reverse transcribed with RevertAid First Strand cDNA Synthesis kit (Thermo Fisher Scientific, USA) according to the instructions in the manual. Quantitative real-time PCR analysis was conducted using the CFX96 Real-Time PCR Detection System (Bio-Rad, USA). Each reaction contained 1 µL of resultant cDNA, 0.5 µL of each primer (10 µmol/L), 0.8 µL of TaqMan probes (10 µmol/L), 8 µL of sterile water and 10 µL of Master Mix (Thermo Fisher Scientific, USA). Amplification was performed as follows: 3 min at 95 °C, followed by 45 cycles of 15 s at 95 °C, 15 s at 58 °C, 15 s at 72 °C. The experiment was run for three biological replicates.

The primers sequences used were as follows: omentin-1, forward 5′-aac-gcc-ttg-tgt-gct-gga-at-3′, reverse 5′-gta-tcc-tcc-tcc-acc-aat-gca-3′, probe 5’-(FAM)-tca-ccg-gat-gta-aca-ctg-ag-(BHQ1)-3’; *FABP4*, forward 5′-gta-cct-gga-aac-ttg-tct-cca-3′, reverse 5′-cat-gcc-agc-cac-ttt-cct-g-3′, probe 5’-(FAM)-aga-agt-agg-agt-ggg-ctt-tgc-(BHQ1)-3’; *RPLP0*, forward 5′-gat-cag-gga-cat-gtt-gct-gg-3′, reverse 5′-gac-ttc-aca-tgg-ggc-aat-gg-3′, probe 5′-(ROX)-caa-taa-ggt-gcc-agc-tgc-tgc-(RTQ2)-3′; *ACTB*, forward 5′-cgt-gct-gct-gac-cga-gg-3′, reverse 5′-aca-gcc-tgg-ata-gca-acg-tac-a-3′, probe 5′-(R6G)-cca-acc-gcg-aga-aga-tga-ccc-aga-t-(BHQ1)-3′. Threshold cycle (Ct) values were obtained and relative gene expression was calculated normalizing to two reference genes (*RPLP0, ACTB*) using the formula according to Vandesompele *et al*. (2002).

### Statistical analysis

Data with normal distribution were expressed as mean ± SD, otherwise as median (lower quartile, upper quartile). Mean values were compared by the Student’s *t* test, while median values were compared using the Mann-Whitney U test. Categorical variables were expressed as percentages and analyzed by a Chi square test. Spearman correlation analysis was performed between mRNA levels and serum concentrations of adipokines and other biochemical markers as well as waist circumference and BMI. In the CAD group extended multivariate linear regression analysis was employed to evaluate relationship between adipokines serum concentration and gene expression level with the following variables: age, gender, waist circumference (as a marker of abdominal obesity), Gensini score, EAT thickness and statin treatment. Covariates were selected on the basis of clinical relevance. In entire cohort variables that explained the largest variability in the patient group and CAD diagnosis as additional factor were analyzed. Statistical analyses were carried out using SPSS 17.0 software (SPSS Inc., Chicago, IL, USA) and R Studio, with p < 0.05 considered as significant.

## Results

### Clinical and biochemical characteristics of the studied groups

Selected clinical and biochemical characteristics of the patients included in this study are summarized in Table 1. Patients with CAD and the representatives of the NCAD group were distributed into subgroups based on the presence of abdominal (central) obesity, thereby dedicating subgroups at increased risk of cardiovascular events for additional analysis. Abdominal obesity was defined as a waist circumference of ≥102 cm for men and ≥88 cm for women according to the American Heart Association/National Heart, Lung, and Blood Institute definition (National Heart, Lung, and Blood Institute, 1998). Overall, the prevalence of abdominal obesity was 51% in the CAD group and 44% in the NCAD group. Lipid levels did not differ between subgroups except serum triglyceride concentration that was higher in obese CAD patients compared to non-obese CAD patients.

**Table 1 -:** Clinical and biochemical characteristics of the studied groups with detailed medications.

	CAD patients N=61		NCAD patients (Control group) N=16	
	Obese N=31	Non-obese N=30	Obese N=7	Non-obese N=9
Age, years	60.6±9.1	61.7±8.1	67.0±3.5	55.8±15.8
Male/female	17/14	24/6	1/6	7/2
Waist circumference, cm	Males: 111 (103-120)^§^ Females: 95 (91-113)^§^	Males: 94 (81-101)^#^ Females: 82 (79-85)	Male: 103 Females: 96 (91-104)	Males: 83 (69-100) Females: 63.5 (58-69)
BMI, kg/m2	31.4±4.5*	25.9±2.7	28.8±4.1	26.7±9.8
EAT thickness, mm	5.3±1.9*^#^	7.2±2.6	6.2±1.4	5.2±2.0
Gensini Score	74 (38-148)	71 (10-202)	-	-
Triglyceride, mmol/L	1.98±1.03*	1.41±0.57	1.86±1.14	1.01±0.48
Total cholesterol, mmol/L	4.43±1.27*	5.21±1.76	5.02±0.88	4.23±1.02
HDL-cholesterol, mmol/L	1.32±0.32	1.25±0.33	1.22±0.22	1.17±0.48
LDL-cholesterol, mmol/L	2.17±1.06*	3.14±1.28	2.94±0.44	2.73±0.63
Fasting glucose, mmol/L	5.95±0.79	5.49±0.92	5.60±1.44	5.41±1.41
Medications, N (%)				
Statins	19 (61%)	16 (53%)	1 (14%)	1 (11%)
Aspirin	18 (58%)	18 (60%)	0 (0%)	0 (0%)
Nitrates	9 (29%)	10 (33%)	0 (0%)	0 (0%)
ACEI/ARB	5 (16%)	4 (13%)	0 (0%)	0 (0%)
Beta-blockers	22 (71%)	25 (83%)	1 (14%)	2 (22%)
Calcium channel blockers	6 (19%)	7 (23%)	0 (0%)	0 (0%)

* p<0.05, obese CAD vs non-obese CAD patients. ^#^ p<0.05, non-obese CAD vs non-obese NCAD. ^§^ p<0.01, obese CAD vs non-obese CAD patients. Abbreviations: ACEI/ARB an angiotensin-converting enzyme inhibitors/ an angiotensin receptor blockers, BMI body mass index, CAD coronary artery disease, EAT epicardial adipose tissue, HDL high density lipoproteins, LDL low density lipoproteins.

### *FABP4* gene expression in EAT and SAT

**Figure 1 -:**
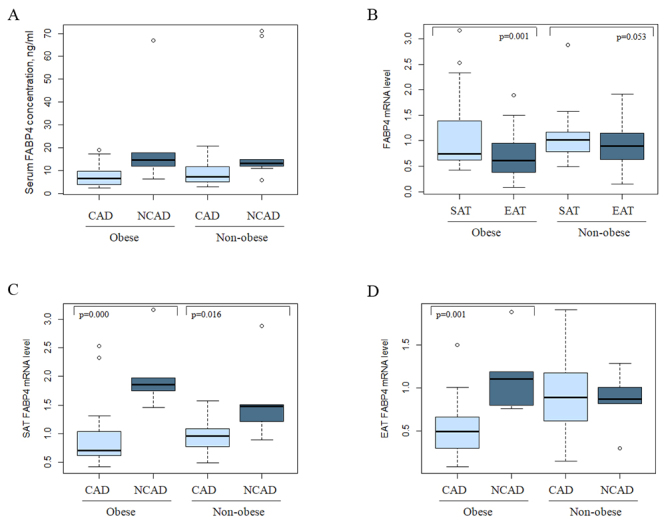
FABP4 serum concentration (A) and gene expression in adipose tissue in obese and non-obese subgroups: comparative analysis of the *FABP4* gene expression in subcutaneous and epicardial fat depots (B); *FABP4* mRNA level in subcutaneous (C) and epicardial adipose tissue (D) in CAD and NCAD individuals.

*FABP4* mRNA level in SAT was higher than in EAT, and the difference was more pronounced in obese individuals (p=0.001) (Figure 1B). SAT *FABP4* mRNA level was lower in CAD patients compared to NCAD individuals in both obese and non-obese subgroups (Figure 1C). EAT *FABP4* mRNA level was reduced only in CAD patients with central obesity as compared with that in the corresponding NCAD individuals (Figure 1D). However, the FABP4 serum concentration did not differ between subgroups (Figure 1A). No association was shown between FABP4 serum concentration and *FABP4* gene expression in adipose tissue. Multivariate regression analysis has shown that FABP4 serum concentrations in CAD patients were strongly predicted by age, sex and waist circumference not by gene expression (Table S1). However, CAD diagnosis was significant predictor for *FABP4* gene expression in SAT independently of age, sex and adiposity (Table S1).

### Omentin-1 gene expression in EAT and SAT

**Figure 2 -:**
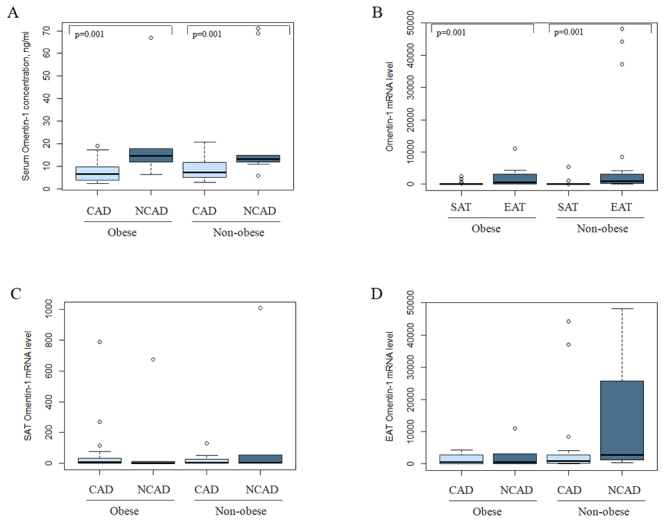
Omentin-1 serum concentration (A) and gene expression in adipose tissue in obese and non-obese subgroups: comparative analysis of the omentin-1 gene expression in subcutaneous and epicardial fat depots (B); omentin-1 mRNA level in subcutaneous (C) and epicardial adipose tissue (D) in CAD and NCAD individuals.

Omentin-1 mRNA level in EAT was markedly increased compared with that in SAT (p=0.001) (Figure 2B). However, no difference in omentin-1 mRNA level was observed in EAT and SAT between CAD and NCAD patients neither in united groups no in subgroups based on obesity status (Figure 2C,D). Neither EAT or SAT omentin-1 mRNA level showed any correlation with omentin-1 serum concentration. At the same time omentin-1 serum concentration was lower in the CAD group than in the NCAD group (p=0.001) (Figure 2A).

Omentin-1 serum concentration and mRNA level in EAT were inversely correlated with waist circumference (r=-0.426, p=0.002 and r=-0.368, p=0.013, respectively) and BMI (r=-0.364, p=0.003 and r=-0.364, p=0.015, respectively). Multivariate regression analysis has shown significant correlation only between omentin-1 serum concentration and waist circumference upon adjustment for age, sex and EAT thickness (β=-0,385, p=0,011 for the CAD group; β=-0,446, p=0,000 for the entire cohort; Table S2). Omentin-1 serum concentration was predicted by CAD diagnosis as well as gender and waist circumference (Table S2).

## Discussion

In the present study, we show that *FABP4* mRNA expression was downregulated in SAT of patients with CAD. At the same time, *FABP*4 mRNA level in EAT was decreased only in obese CAD patients. This finding was unexpected as FABP4 is considered as a pro-inflammatory adipocytokine (Rodríguez-Calvo *et al*., 2017). Studies have shown that increased FABP4 plasma levels predict development of metabolic syndrome and type 2 diabetes mellitus, as well as cardiovascular events in patients with peripheral arterial disease (Xu *et al*., 2007; Ohyama *et al*., 2013; Höbaus *et al*., 2018; Rodríguez-Calvo **et al*.*, 2017). Clinical data and the findings from a FABP4-null mouse study confirmed the role of FABP4 as a key factor involved in the development of obesity-related pathologies in humans (Xu **et al*.*, 2007).

FABP4 is known to be expressed by the macrophages in adipose tissue as well as those from atherosclerotic plaques (Agardh *et al*., 2011; Furuhashi *et al*., 2016). FABP4 was shown to be secreted by macrophages cultured *in vitro* (Furuhashi **et al*.*, 2016). The potential impact of FABP4 on atherosclerosis may be mainly due to value of this molecule in macrophages rather than in adipocytes, as was demonstrated by studies involving bone marrow transplantation (Rodríguez-Calvo *et al*., 2017). Thus, FABP4 locally produced by macrophages from EAT and carotid plaques could contribute to CAD development and progression (Agardh **et al*.*, 2011; Furuhashi **et al*.*, 2016). 

There are only few studies assessing *FABP4* expression level in EAT of CAD patients, with limited sample size ranging from 10 to 16 patients (Vural *et al*., 2008; Sacks *et al*., 2011). In contrast to our data, Vural and colleagues demonstrated higher *FABP4* mRNA levels in EAT and ascending aorta of CAD patients with metabolic syndrome compared with metabolically healthy individuals (Vural **et al*.*, 2008). Ten CAD patients with metabolic syndrome and four controls without metabolic syndrome, who underwent cardiac valvular repair due to aortic stenosis, were examined in their study. Sacks **et al*.* also reported increased *FABP4* mRNA levels in EAT and SAT of 16 patients with severe stable CAD who were overweight as compared to 12 controls (Sacks **et al*.*, 2011). It should be noted that these results should be interpreted with caution as hypoglycemic drugs and statins were shown to reduce FABP4 serum concentration and *FABP4* gene expression (Llaverias *et al*., 2004; Song *et al*., 2010; Hu *et al*., 2012; Furuhashi *et al*., 2015). Furthermore, FABP4 serum and adipose tissue level could be modulated by obesity; hence, we analyzed patients with central obesity and potential metabolic complications separately from non-obese patients. Majority of CAD patients in our study were prescribed statins. Therefore, *FABP4* mRNA reduction in SAT of CAD patients and in EAT of only obese CAD patients may be a consequence of high dose statin treatment. In particular, *FABP4* mRNA was downregulated in EAT of obese CAD patients, consistent with the significant decrease in total and LDL cholesterol levels in this cohort. Intensive treatment may also be the cause of decreased EAT thickness in obese CAD patients (Parisi *et al*., 2019; Raggi *et al*., 2019). Additionally, our results are in agreement with the data of a recent proteomic analysis that showed FABP4 down regulation in EAT of CAD patients (Zhao *et al*., 2019).

Regarding *FABP4* expression and secretion activity of different fat depots, *FABP4* expression was found to be similar in SAT and visceral adipose tissue (VAT) of lean subjects but higher in SAT than in VAT of obese individuals (Fisher *et al*., 2002; Terra *et al*., 2011). EAT is a type of VAT but with specific properties. In our study, *FABP4* mRNA level was higher in SAT than in EAT and this difference was more pronounced in obese individuals. 

At the same time it was shown earlier that SAT and VAT *FABP4* gene expression level did not differ between normally weighing and metabolically healthy obese individuals (Garin-Shkolnik *et al*., 2014; Grzegorczyk *et al*., 2018). However, contradictory results have been published with respect to *FABP4* gene expression in patients with metabolic complications and diabetes. Adipose tissue expression of the *FABP4* gene was shown to be increased in morbidly obese individuals and patients with diabetes, assuming the role of adipose FABP4 in the pathogenesis of insulin resistance and metabolic syndrome (Terra *et al*., 2011; Garin-Shkolnik **et al*.*, 2014). On the contrary, in the study of Queipo-Ortuño and colleagues lean subjects had significantly higher *FABP4* mRNA levels in SAT than overweight, obese and morbidly obese subjects (Queipo-Ortuño **et al*.*, 2012). For morbidly obese subjects a down-regulation of *FABP4* mRNA in VAT was demonstrated, mainly in insulin-resistant patients (Queipo-Ortuño **et al*.*, 2012). FABP4 plasma levels progressively increased with BMI but without any association with *FABP4* gene expression in adipose tissue (Queipo-Ortuño **et al*.*, 2012). 

Apart from well-known value as an adiposity biomarker (Rodríguez-Calvo *et al*., 2017), elevated FABP4 serum concentration on the background of obesity and metabolic syndrome could be also due to it’s high expression in the liver. Hepatic *FABP4* gene expression is increased while adipose tissue expression is decreased in obese mice compared to wild type mice (Queipo-Ortuño *et al*., 2012; Thompson *et al*., 2018). Hepatic expression of the *FABP1*, *FABP4* and *FABP5* genes was shown to be significantly upregulated in morbidly obese patients with insulin resistance (Queipo-Ortuño **et al*.*, 2012). Elevated FABP4 serum concentrations and higher gene expression in the liver were reported in patients with hepatic pathology (Graupera *et al*., 2017; Thompson **et al*.*, 2018). These facts indicate that adipose tissue gene expression could not fully predict FABP4 serum concentration in obese patients with metabolic complications what we also show in our study.

In the present study, omentin-1 serum concentrations were decreased in CAD patients. Previous meta-analyses demonstrated that serum omentin-1 concentration was independently and negatively associated with CAD and reduced in obese individuals (Agasthi *et al*., 2015; Arab *et al*., 2020). Researchers who performed studies on patients with type 2 diabetes mellitus suggest that decreased omentin-1 level may be an independent risk factor for arteriosclerosis and carotid plaque formation in these patients (Yoo *et al*., 2011; Nishimura *et al*., 2019; Biscetti *et al*., 2020). Low omentin-1 serum concentration was associated with acute coronary syndrome, stable angina and ischemic stroke (Shibata *et al*., 2011; Zhong *et al*., 2011; Yue *et al*., 2018). Other investigators reported that elevated omentin-1 plasma level predicts cardiovascular events (Menzel *et al*., 2016; Saely *et al*., 2016). At the same time *in vitro* studies of exogenous omentin-1 expression proved the cardioprotective functions of omentin-1: omentin-1 improved insulin sensitivity and anti-inflammatory activity of cultured EAT and SAT tissue samples of patients with cardiovascular disease and reduced atherosclerotic plaque formation in an ApoE-deficient animal model of atherosclerosis (Hiramatsu-Ito *et al*., 2016; Fernández-Trasancos *et al*., 2017).

Contradictory results have been published earlier on the omentin-1 gene expression in EAT of patients with CAD. While Harada and colleagues showed that omentin-1 gene expression was upregulated in CAD patients, Du and colleagues demonstrated decreased omentin-1 gene expression in EAT of CAD patients (Du **et al*.*, 2016; Harada **et al*.*, 2016). Both studies were conducted with limited sample sizes and didn’t include overweight CAD patients and controls. In our study we included a larger number of patients with CAD and without CAD (NCAD) with normal body weight or with obesity. We demonstrated that omentin-1 mRNA levels in EAT and SAT did not differ significantly between CAD and NCAD groups including when compared separately in subgroups based on obesity status. These findings point out that omentin-1 gene expression in EAT and SAT is not associated with CAD. 

We also confirm that omentin-1 expression is upregulated in EAT as compared with SAT, as previously demonstrated (Fain *et al*., 2008, Gaborit *et al*., 2015). Summarizing our results, we conclude that SAT may be inactive in omentin-1 secretion. Omentin-1 predominantly is synthesized by VAT, not by SAT, and is highly expressed in epicardial fat (de Souza Batista **et al*.*, 2007; Fain **et al*.*, 2008, Gaborit **et al*.*, 2015). As omentin-1 is secreted by VAT, most of circulating omentin-1 may be of omental fat origin. We can conclude EAT and SAT gene expression could have no significant effect on omentin-1 serum level.

In conclusion, our study demonstrated reduced omentin-1 serum concentrations in patients with CAD. Thus we confirm the protective role of serum omentin-1 demonstrated in previous studies. We also highlight that *FABP4* gene expression in SAT is reduced in CAD patients and proposed that decreased EAT and SAT *FABP4* mRNA levels could be associated with CAD development in obese individuals or statin treatment. 

There are several limitations to this study: limited sample size of the control group; gender differences of degree of obesity in the studied groups; additionally the drug usage effect in CAD patients could not be excluded. Further prospective studies are needed to evaluate whether *FABP4* mRNA levels in EAT and SAT could be used as predictors of CAD development.

## Supplementary material

The following online material is available for this article:

Table S1 ‒ Multiple regression analysis between age, sex, abdominal obesity and FABP4 serum concentration and mRNA level in epicardial and subcutaneous adipose tissue.

Table S2 ‒ Multiple regression analysis between age, sex, abdominal obesity and omentin-1 serum concentration and mRNA level in epicardial and subcutaneous adipose tissue.
