# Supplementary material for: FABP4 and omentin-1 gene expression in epicardial adipose tissue from coronary artery disease patients
Source: Genet Mol Biol. 2021 Sep 29;44(4):e20200441. doi: 10.1590/1678-4685-GMB-2020-0441 (PMC8485182; doi:10.1590/1678-4685-GMB-2020-0441)
Supplement: Table S1 ‒ [file 1415-4757-GMB-44-4-e20200441-s1.pdf]

Genetics and Molecular Biology
https://doi.org/10.1590/1678-4685-GMB-2020-0441

Supplementary Material to "*FABP4* and omentin-1 gene expression in epicardial adipose tissue from coronary artery disease patients"

Table S1 - Multiple regression analysis between age, sex, abdominal obesity and FABP4 serum concentration and mRNA level in epicardial and subcutaneous adipose tissue.

	Adjusted R^2 and p value for model<br>β-Coefficients (p value)		
Parameters	FABP4 serum concentration	FABP4 SAT mRNA level	FABP4 EAT mRNA level
A. Analysis in the CAD group			
**Model 1**	R^2=0,223, p=0,000	R^2=0,054, p=0,100	R^2=-0,040, p=0,763
Age	**0,338 (0,005)**	0,154 (0,282)	0,038 (0,819)
Gender (woman)	**0,329 (0,006)**	0,225 (0,119)	0,111 (0,512)
**Model 2**	R^2=0,210, p=0,003	R^2=0,141, p=0,032	R^2=-0,080, p=0,888
Age	**0,276 (0,039)**	0,131 (0,395)	-0,057 (0,763)
Gender (woman)	**0,367 (0,009)**	**0,329 (0,044)**	-0,096 (0,621)
+ Waist circumference	**0,369 (0,009)**	-0,130 (0,408)	-0,132 (0,502)
**Model 3**	R^2=0,177, p=0,050	R^2=-0,122, p=0,880	R^2=-0,081, p=0,676
Age	0,255 (0,134)	-0,109 (0,617)	-0,187 (0,425)
Gender (woman)	0,424 (0,024)	-0,012 (0,961)	0,031 (0,901)
Waist circumference	0,372 (0,033)	-0,077 (0,729)	-0,281 (0,240)
+ Gensini score	0,070 (0,697)	-0,161 (0,501)	0,159 (0,531)
**Model 4**	R^2=0,204, p=0,017	R^2=-0,107, p=0,896	R^2=-0,086, p=0,720
Age	0,236 (0,112)	-0,126 (0,531)	-0,126 (0,568)
Gender (woman)	**0,320 (0,039)**	0,017 (0,933)	-0,128 (0,575)
Waist circumference	**0,351 (0,033)**	-0,146 (0,518)	-0,267 (0,266)
+ Statins	0,222 (0,147)	-0,059 (0,777)	0,248 (0,282)

Parameters	FABP4 serum concentration	FABP4 SAT mRNA level	FABP4 EAT mRNA level
**Model 5**	R^2=0,163, p=0,039	R^2=0,246, p=0,017	R^2=-0,084, p=0,740
Age	0,306 (0,054)	0,228 (0,158)	-0,133 (0,545)
Gender (woman)	0,346 (0,057)	**0,476 (0,018)**	-0,195 (0,449)
Waist circumference	0,307 (0,066)	-0,144 (0,404)	-0,173 (0,456)
+ EAT thickness	-0,015 (0,934)	**0,414 (0,035)**	-0,351 (0,185)
**Model 6**	R^2=0,153, p=0,123		
Age	0,326 (0,105)	-	-
Gender (woman)	0,032 (0,884)	-	-
Waist circumference	0,335 (0,123)	-	-
+ SAT mRNA level	0,388 (0,098)	-	-
+ EAT mRNA level	0,071 (0,721)	-	-
**Model 7**	R^2=0,242, p=0,000	R^2=0,064, p=0,040	R^2=-0,035, p=0,632
Age	**0,310 (0,010)**	0,183 (0,187)	0,027 (0,877)
Gender (woman)	**0,397 (0,001)**	0,211 (0,134)	0,165 (0,340)
+ serum omentin-1	-0,065 (0,579)	**0,288 (0,040)**	-0,157 (0,360)
**Model 8**	R^2=0,122, p=0,008	R^2=0,186, p=0,003	R^2=-0,101, p=0,884
Age	0,188 (0,173)	0,203 (0,154)	-0,097 (0,623)
Gender (woman)	**0,374 (0,008)**	0,279 (0,066)	-0,033 (0,867)
Waist circumference	0,272 (0,050)	-0,134 (0,378)	-0,094 (0,641)
+ serum omentin-1	-0,065 (0,579)	**0,454 (0,003)**	-0,201 (0,334)
B. Analysis in the entire cohort			
**Model CAD**	R^2=0,171, p=0,002	R^2=0,355, p=0,000	R^2=-0,004, p=0,443
Age	0,179 (0,126)	0,261 (0,996)	-0,023 (0,888)
Gender (woman)	**0,325 (0,006)**	0,255 (0,912)	0,024 (0,883)
Waist circumference	**0,381 (0,004)**	-0,014 (0,864)	0,048 (0,773)
+ CAD	**-0,249 (0,049)**	**-0,606 (0,000)**	0,306 (0,072)

Abbreviations: BMI – body mass index, CAD coronary artery disease, EAT – epicardial adipose tissue
