# Supplementary material for: FABP4 and omentin-1 gene expression in epicardial adipose tissue from coronary artery disease patients
Source: Genet Mol Biol. 2021 Sep 29;44(4):e20200441. doi: 10.1590/1678-4685-GMB-2020-0441 (PMC8485182; doi:10.1590/1678-4685-GMB-2020-0441)
Supplement: Table S2 ‒ [file 1415-4757-GMB-44-4-e20200441-s2.pdf]

Genetics and Molecular Biology
https://doi.org/10.1590/1678-4685-GMB-2020-0441

Supplementary Material to "*FABP4* and omentin-1 gene expression in epicardial adipose tissue from coronary artery disease patients"

Table S2 - Multiple regression analysis between age, sex, abdominal obesity and omentin-1 serum concentration and mRNA level in epicardial and subcutaneous adipose tissue.

	Adjusted R^2 and p value for model<br>β-Coefficients (p value)		
Parameters	Omentin-1 serum concentration	Omentin-1 SAT mRNA level	Omentin-1 EAT mRNA level
A. Analysis in the CAD group			
**Model 1**	R^2=0,009, p=0,292	R^2=-0,047, p=0,980	R^2=0,011, p=0,305
Age	0,118 (0,372)	-0,004 (0,982)	-0,058 (0,727)
Gender (woman)	0,150 (0,259)	-0,030 (0,847)	0,256 (0,126)
**Model 2**	R^2=0,089, p=0,064	R^2=-0,079, p=0,944	R^2=-0,020, p=0,508
Age	0,031 (0,830)	0,007 (0,968)	-0,015 (0,937)
Gender (woman)	0,253 (0,083)	-0,110 (0,551)	0,294 (0,148)
+ Waist circumference	-0,214 (0,142)	-0,012 (0,949)	0,052 (0,789)
**Model 2A**	R^2=0,042, p=0,145	R^2=-0,071, p=0,995	R^2=0,005, p=0,372
Age	0,098 (0,451)	-0,071 (0,985)	-0,049 (0,766)
Gender (woman)	0,153 (0,240)	-0,031 (0,845)	0,241 (0,151)
+ BMI	-0,222 (0,088)	0,029 (0,855)	-0,140 (0,382)
**Model 3**	R^2=-0,077, p=0,786	R^2=-0,110, p=0,836	R^2=0,147, p=0,178
Age	-0,077 (0,786)	-0,012 (0,954)	-0,206 (0,368)
Gender (woman)	-0,058 (0,760)	-0,049 (0,827)	-0,181 (0,448)
Waist circumference	0,161 (0,435)	-0,056 (0,797)	-0,525 (0,028)
+ Gensini score	-0,049 (0,812)	0,229 (0,309)	-0,210 (0,385)

Parameters	Omentin-1 serum concentration	Omentin-1 SAT mRNA level	Omentin-1 EAT mRNA level
**Model 4**	R^2=-0,063, p=0,783	R^2=-0,127, p=0,930	R^2=0,100, p=0,206
Age	-0,092 (0,593)	0,004 (0,983)	-0,240 (0,253)
Gender (woman)	0,183 (0,294)	-0,125 (0,551)	-0,204 (0,352)
Waist circumference	-0,062 (0,739)	-0,102 (0,654)	-0,490 (0,034)
+ Statins	-0,039 (0,825)	0,156 (0,483)	0,165 (0,420)
**Model 5**	R^2=0,236, p=0,003	R^2=-0,047, p=0,610	R^2=0,008, p=0,407
Age	0,022 (0,877)	0,211 (0,313)	0,164 (0,456)
Gender (woman)	**0,297 (0,046)**	-0,257 (0,272)	0,427 (0,073)
Waist circumference	**-0,385 (0,011)**	-0,074 (0,736)	0,132 (0,554)
+ EAT thickness	0,072 (0,670)	-0,175 (0,474)	0,283 (0,244)
**Model 6**	R^2=-0,103, p=0,736		
Age	0,126 (0,598)	-	-
Gender (woman)	0,044 (0,855)	-	-
Waist circumference	-0,241 (0,295)	-	-
+ SAT mRNA level	-0,150 (0,496)	-	-
+ EAT mRNA level	-0,054 (0,813)	-	-
**Model 7**	R^2=-0,014, p=0,541	R^2=-0,062, p=0,933	R^2=0,001, p=0,668
Age	0,139 (0,332)	-0,031 (0,850)	0,001 (0,996)
Gender (woman)	0,160 (0,282)	-0,076 (0,665)	0,290 (0,097)
+ serum FABP4	-0,087 (0,579)	0,113 (0,534)	-0,144 (0,443)
**Model 8**	R^2=0,093, p=0,081	R^2=-0,104, p=0,961	R^2=-0,053, p=0,668
Age	-0,035 (0,814)	0,044 (0,824)	0,006 (0,978)
Gender (woman)	0,135 (0,398)	-0,077 (0,699)	0,305 (0,148)
Waist circumference	-0,293 (0,063)	0,015 (0,938)	0,070 (0,736)
+ serum FABP4	0,228 (0,163)	-0,101 (0,627)	-0,058 (0,777)
B. Analysis in the entire cohort			
**Model 9**	R^2=0,355, p=0,000	R^2=0,157, p=0,040	R^2=-0,061, p=0,748
Age	0,021 (0,855)	**0,351 (0,034)**	-0,019 (0,918)
Gender (woman)	**0,366 (0,002)**	-0,114 (0,509)	0,238 (0,206)
Waist circumference	**-0,446 (0,000)**	**-0,358 (0,028)**	0,026 (0,882)
EAT thickness	-0,021 (0,860)	-0,242 (0,131)	0,122(0,494)

Parameters	Omentin-1 serum concentration	Omentin-1 SAT mRNA level	Omentin-1 EAT mRNA level
**Model CAD1**	R^2=0,376, p=0,000	R^2=-0,081, p=0,982	R^2=-0,036, p=0,650
Age	-0,023 (0,822)	0,067 (0,684)	-0,055 (0,742)
Gender (woman)	**0,303 (0,004)**	-0,084 (0,634)	0,232 (0,174)
Waist circumference	**-0,276 (0,014)**	-0,065 (0,701)	-0,026 (0,874)
+ CAD	**-0,312 (0,006)**	0,021 (0,905)	0,149 (0,366)
**Model CAD2**	R^2=0,420, p=0,000	R^2=0,140, p=0,069	R^2=-0,061, p=0,712
Age	0,020 (0,868)	0,365 (0,031)	-0,015 (0,934)
Gender (woman)	**0,323 (0,004)**	-0,143 (0,431)	0,273 (0,156)
Waist circumference	**-0,320 (0,009)**	-0,321 (0,066)	-0,040 (0,832)
EAT thickness	0,019 (0,870)	-0,227 (0,164)	0,104 (0,564)
+ CAD	**-0,305 (0,013)**	-0,104 (0,564)	0,188 (0,328)

Abbreviations: BMI – body mass index, CAD coronary artery disease, EAT – epicardial adipose tissue
